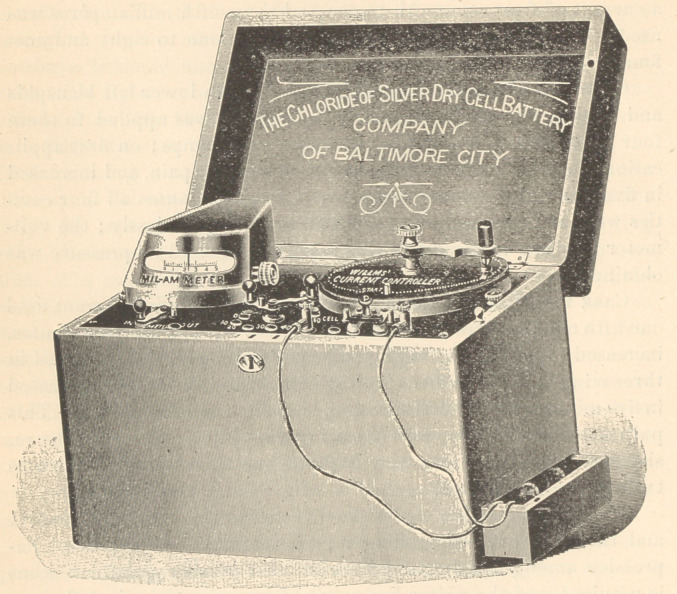# The Advantages of Cataphoresis in Dental Operations

**Published:** 1896-06

**Authors:** Peter Brown

**Affiliations:** Montreal


### THE ADVANTAGES OF CATAPHORESIS IN DENTAL OPERATIONS.

BY PETER BROWN, L.D.S., MONTREAL.

THE medication of dentine for the alleviation of pain usually accompanying the operation of filling teeth, has received a large share of attention from the dental profession, and no other tissue in the human body has been so difficult to temporarily anæsthetize without danger of destruction, as the fibrillæ in the dentine.

On the introduction of cocaine some years ago as a local anæsthetic, the dental world hailed its advent with much anticipation of a successful obtundent, but, much to our disappointment, it was found to be a failure as far as its effect on dentine was concerned.

More recently experiments were conducted with a mild current of electricity with a view to forcing this alkaloid to enter the dentine by the action known as cataphoresis, it is also known as electrical osmosis, or anodal diffusion, and is the passing of a medicinal substance such as quinine, strychnine, cocaine, or iodine, through organic tissues in the direction of the flow of the current, that is, from the anode to the cathode.

This property of electricity has been known for a long time, but its application to this treatment is of recent date. Its success in this direction has been so abundantly proved by reputable and competent practitioners that its use will soon become as universal as the dental engine.

Following is a report of a number of cases taken from every-day practice with records of methods employed for the application and graduating of the current, and the quantity and the pressure of the circuit at the time of passing through the tooth under treatment.

Case I.—Master T., aged twelve. Right lateral incisor, servical cavity extending across the labial and around to the median approximal surface; this was a very sensitive cavity as these cavities usually are; after the dam was applied and the cavity wiped dry, a saturated solution of cocaine was placed in it on a small piece of cotton, a bicuspid clamp to which was attached a piece of brass wire with a platinum tip was adjusted to the bicuspid of the same side and outside the dam; this wire was not in electrical contact with the clamp, it was fixed there by being vulcanized to it, there being a thin layer of rubber between the wire and the clamp; it did not matter if the clamp cut through the rubber dam, the battery wire was secured to the electrode by a spring clip; the current being turned on, the milammeter indicated one-fifth of a milliampère and the voltmeter one and three-eighths volts before the patient gave evidence that the current was felt; in five minutes the current was increased to two-fifths of a milliampère and the voltage to two and one-eighth; in five minutes more the current was increased to two-fifths of a milliampère at a pressure of four and one-fourth volts; this was allowed to remain for three minutes, when the cavity was excavated without pain until nearly the end, when a second application was made and a maximum quantity of one and three-tenths milliampères was indicated at a potential of eight and one-fourth volts.

Case II.—Miss M. Right superior first molar; pulp exposed and patient suffering toothache at time of treatment; current applied same as in previous case and following readings noted; on first evidence of the current being felt a current of one-twentieth milliampère was indicated at a potential of three-eighths of a volt; in ten minutes the current was increased to one-tenth milliampère at one volt; in five minutes more to one-fifth milliampère at one and one-half; after ten minutes' treatment at this rate the pulp was found to be completely anæsthetized, and was removed in the usual manner without pain. This case was remarkable from the small quantity of the current used, the maximum quantity being one-fifth and the maximum voltage two and seventh-eighths.

Case III.—Mr. B. Pulp exposed in lower molar; preparation same as in previous cases; current indicated at first contact one-tenth milliampère, increased in five minutes to one-fifth, in three minutes to two-fifths, within the following five minutes to three-fifths; within twenty minutes from first application the electrodes were removed and the cavity excavated with a No. 8 round bur in right angle high pressure, completely removing the coronal portion of the pulp; the roots were still sensitive, requiring a second application for five minutes, when the roots were cleaned out as thoroughly as these roots can be cleaned. In the second application a current of from one-tenth to one and three-fifth milliampères was used. The voltage varied in this case from one to eight and one-fourth.

Case IV.—Mrs. T. Approximal cavities in lower left bicuspids and canine, four cavities in all. The current was applied to these four cavities at the same time by using two clamps; on first application a current of three-fifths was used without pain, and increased in five minutes to one milliampère; in twelve minutes all four cavities were anæsthetized and prepared for filling painlessly; the voltmeter was not in circuit in this case, so no record of pressure was obtained.

Case V.—Miss W. Left lower molar exposed pulp; current used one-fifth milliampères at two and one-fourth volts; time five minutes, increased to two-fifths; voltage nine and seven-eighths, increased in three minutes to three-fifths; voltage fifteen and one-fourth, increased in five minutes to four-fifths; voltage nineteen and five-eighths. This pulp was completely anæsthetized in twenty-five minutes. The resistance in this case was very high, at one time being as much as twenty thousand ohms.

Too much attention cannot be directed to the source of current and the means for controlling it; there seems to be a general impression among the dental profession that a volt-regulator is what is required, and the writer has received many inquiries in reference to using street circuits and dynamos and volt-selectors, etc. It should be borne in mind that it is the quantity of current or amperage that does the work; the pressure or voltage merely overcomes the resistance and forces the current through the circuit.

In order to do this satisfactorily and with a minimum of pain or disagreeable sensation to the patient, we must have a steady and reliable source of current and a reliable rheostat to graduate this current. The rheostat, or regulator, or controller, as it may be termed, should be capable of varying the pressure and quantity of the current without sudden interruptions or sudden increase of current.

The most satisfactory apparatus the writer has used yet is that illustrated on page 368. Here we have twenty-five cells of dry battery which will last a dentist for cataphoric work for at least two years, and then may be renewed at less than one-third the original cost of battery; they are superior to the storage-battery, as they require no special care or attention. In the same case will be found a "Willms dry current controller," with switch-board or cell-selector.

When desired, a milammeter may be added to the outfit, and will be found a very desirable addition, as one can always be assured that the current is passing through the patient under treatment.

*Medicaments.*—The writer has experimented with varying success with "aconitine," using a specially-made triturate of one one-hundredth grain. This drug was suggested by Dr. Charles Brewster, of Montreal, who used it for obtunding sensitive dentine thirty years ago.

In two cases tried it was successful, but failed in others to produce anæsthesia as thoroughly as cocaine.

In using the guiacol cocaine solution, it was found in the writer's experience that no better results were obtained by this combination; it was also ascertained that guiacol was not a preservative of cocaine and the solution decomposed. The odor of guiacol is also very objectionable. A saturated solution of cocaine is the best agent to employ, made just as used by saturating about one-sixth or oneeighth grain with enough water to dissolve it, and absorbing it on cotton sufficient to fill the cavity under treatment.

An improved method of clamp for holding the electrode in the cavity is to have a small set screw attached to the clamp and insulated from it by a rubber washer; then, if the clamp comes in contact with the tooth, there is no circuit established outside where it is not wanted.

The following case shows the advantage of a milammeter. A clamp was attached to a lower first molar outside the dam; a current of three-tenths milliampère was indicated in the meter. A few minutes later on, glancing at the milammeter, a current of three and one-half milliampères was indicated. This showed a short circuit somewhere; it was found in a hole in the dam, made by the point of the clamp.

There is good scope for improvements in methods of attachment of electrodes; they must be readily attached, capable of adjustment in any direction, and allow the patient freedom in moving the head, and to some extent the lower jaw.

A good plan for the negative pole attachment is an arrangement shaped like a horseshoe and placed over the wrist. An adjustable screw may be put through one side of it and bear on a disk which would press down on the wrist; this would give the greatest freedom to use the hands to hold a paper or magazine which the patient might read while under treatment. Moist lintine should be placed next to the skin, as the moist surface makes a good conductor and lessens resistance. So far no bad results have been noted from this treatment. Cocaine has no local destructive action on nerve tissue that we are at present aware of, though it has a very destructive action on nerve-cells when taken systemically and for a continued period.

The quantity of electricity used is so small and so mild in strength that it can have no effect as a tissue-destroyer. Now that the efficacy of this treatment has been established, we can only hope that no unexpected results will crop up to mar our anticipation of painless operations on the teeth.

## Figures and Tables

**Figure f1:**